# Response to unexpected social inclusion: A study using the cyberball paradigm

**DOI:** 10.3389/fpsyt.2022.911950

**Published:** 2022-08-03

**Authors:** Rosa-Marie Groth, Winfried Rief

**Affiliations:** Department of Clinical Psychology and Psychotherapy, Philipps-University of Marburg, Marburg, Germany

**Keywords:** expectation, fundamental needs, expectation change, social inclusion, cognitive immunization, cognitive behavioral therapy, Major Depressive Disorder (MDD)

## Abstract

**Background:**

Dysfunctional expectations are considered core characteristics of Major Depressive Disorder (MDD) and should be focused in psychotherapy. Dysfunctional expectations are especially pronounced in the interpersonal area (social expectations). In the present study, we examine the effect of unexpected social inclusion (expectation violation) on the change of generalized and specific depression-typical social expectations.

**Method:**

We conducted an online study to investigate the impact of social inclusion after a period of social exclusion (unexpected social inclusion) on social expectation change (sample size 144) in a non-clinical sample. Depressive symptoms were assessed *via* self-reporting. Participants took part in two rounds of the online ball-game Cyberball. In the first round, all participants were socially excluded by their two co-players (acquisition of negative social expectations). In the second round, participants were either once more excluded (expectation confirmation) or included equally (expectation violation) by the same co-players. Specific and generalized social expectations were assessed after each round.

**Results:**

Specific and generalized social expectations increased following expectation violation. Even though depressive symptoms were related to lower levels of social expectations, we found that depressive symptoms did not moderate expectation change after positive expectation violations.

**Conclusions:**

In the present experimental setup including the use of the online ball-game Cyberball, the establishment and change of social expectations can be experimentally manipulated. Under the given circumstances and in a non-clinical sample, negative expectations can be updated after unexpected positive experiences regardless of the number of depressive symptoms. The results are discussed in the context of current models of Major Depressive Disorder (MDD), expectation change, and cognitive behavioral therapy.

## Introduction

Mood Disorders are a group of mental diseases that are primarily affecting feelings, self-motivated behavior and drive (1). Above all, the term Mood Disorder includes Major Depression Disorder (MDD) and Bipolar Disorder. Major Depression Disorder (MDD) is one of the most common and most handicapping of mental disorders worldwide (2, 3). Diagnostic criteria are written down in the ICD-10 (69) and the DSM-V (4). According to the ICD-10, a diagnosis of a MDD requires the presence of at least four related symptoms for a minimum of 14 days. Core symptoms are dejected mood, loss of pleasure or interest and reduced drive.

There are multiple theories about the pathophysiology of Major Depressive Disorder (MDD), for example from genetic and environmental research (5) as well as multiple fields in neuroscience (6). Several brain abnormalities have been associated with Major Depressive Disorder (MDD) (7–10), especially changes in cortical and limbic brain regions. These brain regions are not only essential for emotional experience, learning and emotional regulation (11), but also to align one's own behavior according to adaptive goals (11, 12). Among these studies, especially the abnormal functioning of the ventromedial prefrontal cortex (vmPFC) excels (9). The human vmPFC plays a major role in pavlovian fear conditioning (13), which in its adaptive form, from an evolutionary perspective, is increasing the individuals chances to survive (14). Lesions in the vmPFC, on the other hand, lead to impaired extinction learning (15, 16), which can result in a reduced ability to adapt to actually harmless situations (17) and support depression-like behavior, for example learning from rewards and emotional learning capacity (18).

In psychotherapeutic practice, there is increasing, cross-procedural agreement that it is a core process of psychotherapy to enable and motivate patients to expose themselves to feared situations in order to gain new, expectation violating experiences and create less-restricted, value-driven lives [e.g., (19)]. In the case of a patient with Major Depressive Disorder (MDD), this could mean helping the patient to get back out among people in order to have new experiences that contradict their negative beliefs (20, 21) (e.g., “When I am around people, I will be rejected”), which often derive from negative childhood experiences (22). Therefore, therapy strives for an extinction of the previous learned belief and emotion (e.g., fear) by repeated exposure to situations where these fears are not met. Considerable research has emphasized the role of the interaction of the medial prefrontal cortex and the amygdala to fear extinction (17). The failure of people with Major Depressive Disorder (MDD) to behave according to desirable goals are core symptoms of the disease. It is conceivable that abnormalities in the ventromedial prefrontal cortex provides important contributions to these symptoms (23).

### Cognitive deficits in clinical practice and social expectations

Studies have suggested these deficits are especially pronounced in the interpersonal area (24–27). It is therefore the goal of modern psychotherapies that patients change their behavior in a way that positive interactions with other people become more likely (28, 29). However, expectation violations do not automatically lead to the dysfunctional assumption being revised (30, 31). The concept of “cognitive immunization” has been introduced to explain the maintenance of dysfunctional expectations despite expectation-disconfirming experiences (31, 32), which can be discussed as a reason for relapsing into old behaviors—for example, social withdrawal. How people with mental illness deal with positive experiences that violate their dysfunctional negative expectations is therefore of immense importance (31, 33–35). Only a few studies have addressed the shift in depression-typical social expectations following positive and rewarding social experiences [e.g., (36, 37)]. This may be due to a lack of feasible and readily interpretable measures of interpersonal expectation change. Therefore, the aim of this experimental study was to systematically examine the establishment and change of social expectations following unexpected social inclusion.

The second aspect we wanted to focus on were the feelings that arise in social situations, and therefore the fulfillment of reflexive needs in social situations. Social exclusion brings out feelings of pain (38) and threatens our basic needs—the feeling of belonging, self-esteem, meaningful existence, and control (71). A recent study (39) reported that depressive symptoms were not only negatively associated with need satisfaction, but also associated with a slower recovery from social exclusion. Furthermore, studies have shown that a threat or default of those needs can motivate performance in non-depressed individuals (40). This behavioral aspect of emotion regulation is a major reason why this process is considered a core process and a major component of therapies across disorders (41). Therefore, we wanted to manipulate both social expectations and the feelings and needs associated with social inclusion and exclusion, conceptualized as reflexive needs (71). Furthermore, the paradigm was expected to allow us to experimentally manipulate the degree of social inclusion and exclusion in order to measure the effect of unexpected social inclusion.

### Cyberball

We opted for the Cyberball paradigm (42), Cyberball being an online ball-tossing game frequently used to study the effects of social inclusion or ostracism. Again, however, the majority of studies have dealt with social exclusion [for a review see Hartgerink et al. (43)]. Seidl et al. (44) as well as Jobst et al. (45) reported, that patients with chronic depression reacted more strongly to social exclusion during Cyberball game compared to healthy controls. Only a comparatively small number have dealt with explicit social inclusion, even though also the results conclude that subjects with Major Depressive Disorder (MDD) seem to have deficits in processing positive social stimuli (37). The combination of both (i.e., periods of social exclusion and inclusion in the same participant) has also tended to be neglected. However, for psychotherapy to be effective, this individual response to new experiences in the same context is of great importance. As far as we know, this is the first study to systematically examine the effect of social inclusion after a period of social exclusion using the Cyberball paradigm.

Our online experiment was built around two rounds of Cyberball, with the participant being asked to toss the ball in a game with two co-players. According to the cover story, the co-players were two other participants, whereas in reality, they were computer-generated. In the first round, we aimed to target and induce depression-typic social expectations (“I will be rejected”) by ostracizing the participant. Ostracizing meant that the participant was not passed the ball at all after two ball contacts at the beginning of the game. In the second round, the participants were either once more ostracized (expectation confirmation) or included (expectation violation) by the same co-players. Social expectations and reflexive needs were both assessed after each round. The main goal of the study was to examine the influence of this manipulation on expectation change as well as the role of depressive symptoms in a non-clinical sample. The side aspect was the shift in reflexive need fulfillment related to social inclusion and exclusion. With regard to the literature cited above, our main hypotheses were that:

1. the present experimental setup with the online ball-game Cyberball would a be suitable paradigm to establish and change specific and generalized social expectations and the prevalence of depressive symptoms would moderate the effect of expectation violation caused by unexpected social inclusion.
2. Additionally, we expected a similar pattern for the shift in feelings associated with social inclusion and exclusion, conceptualized as four reflexive needs—control, belonging, meaningful existence, and self-esteem.

## Methods

**Figure 1:**
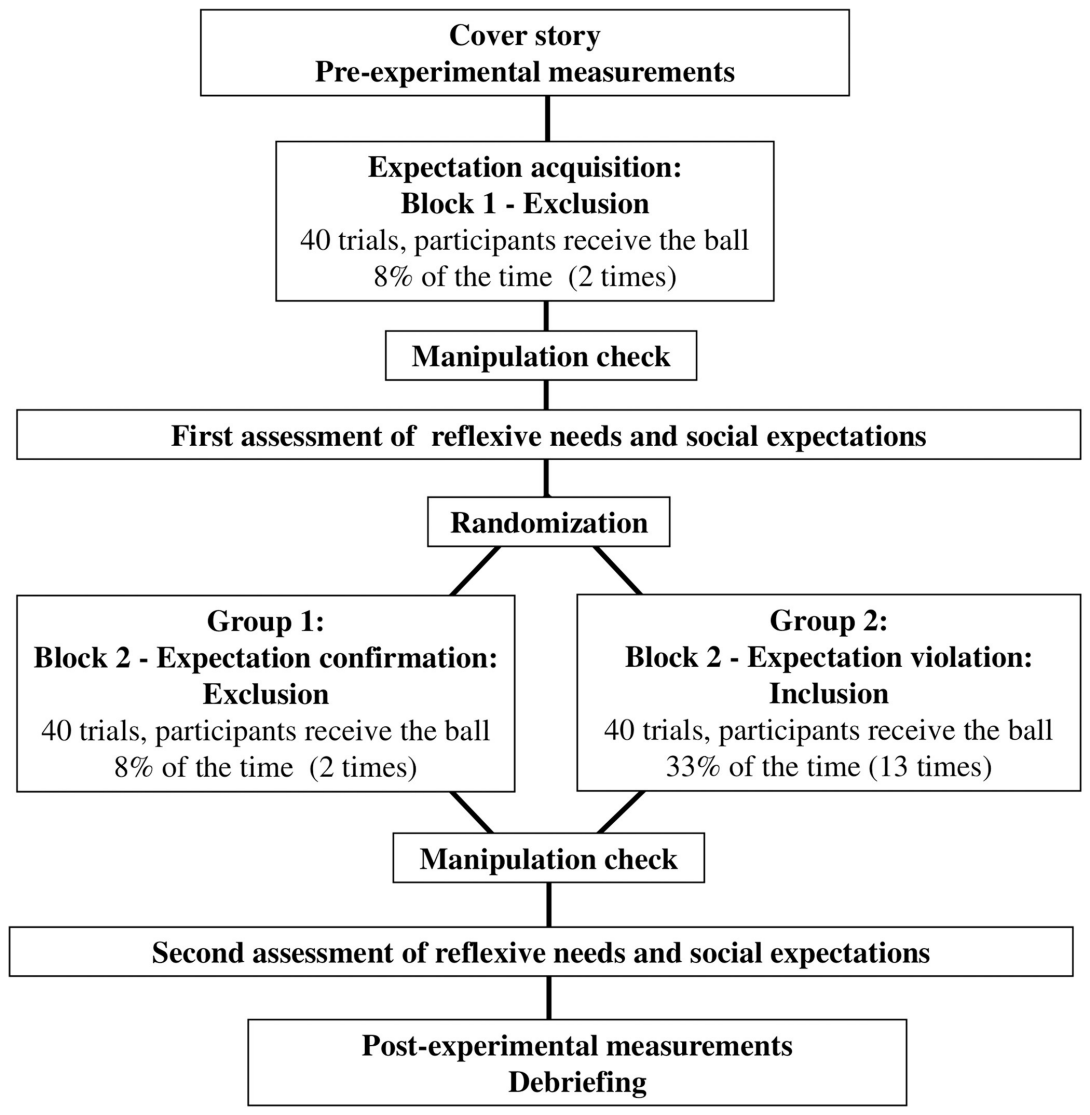
Study procedure. A cover story and pre-experimental measurements marked the beginning of the study. Negative social expectations were built up in the first round of the online ballgame Cyberball (40 trials, participants received the ball twice). After the first round, we performed a manipulation check. Social expectations and reflexive needs were assessed for the first time. The second round then followed. After randomization, the participants either experienced social exclusion (Group 1—expectation confirmation, 40 trials, unchanged rate of ball contact) or unexpected social inclusion (Group 2—expectation violation, 40 trials, participants receive the ball 13 times). The manipulation check (“Assuming that the ball should be thrown to each person equally [33% in that case], what percentage of the throws did you receive?”) and the participant's reflexive needs and social expectations were assessed again. Thereafter, post-experimental measurements and debriefing marked the end of the study.

The experiment was conducted online. The main part of the study consisted of two rounds of the online ball game Cyberball (42). In the first round, the participants experienced social exclusion, regardless of experimental condition, in order to build up negative social expectations. In the second round, the participants either were once again excluded (Group 1, expectation confirmation) or unexpectedly included (Group 2, expectation violation). Reflexive needs and social expectations were both assessed after each round. The procedure of the study is illustrated in Figure 1.

### Ethics

The study was approved by the local ethics committee of Philipps University Marburg (Reference Number 2021-44k) and was conducted in accordance with the ethical standards laid down in the Declaration of Helsinki and its amendments. All participants were treated in accordance with the ethical guidelines of the German Psychological Society and gave informed consent.

### Sample

An a-priori power analysis (expected f = 0.15; alpha = 0.05; power (1-β error prob) = 0.95) indicated a sample size of 148 participants. Only participants who completed the study were included in the analysis. The total sample consisted of 155 participants. Participants were randomly assigned to one of the two experimental conditions (expectation violation vs. expectation confirmation). They were recruited *via* online forums, email lists, and social media. Inclusion criteria were sufficient German language skills, no visual impairment, a stable Internet connection, no participation in previous studies in our department using the Cyberball paradigm, aged at least 18 years, and having either no current or previous mental disorders or a current diagnosis of a Major Depressive Disorder (MDD). The participants received one course credit or the chance to win a tablet (worth approximal €200) as an incentive for their participation. We excluded participants if they failed the manipulation check. After exclusion, 144 participants remained in the final analysis; Table 1 provides the demographic data for these.

**Table 1:** Sociodemographic characteristics, baseline characteristics and self-rating of the self-rating on social expectations of the sample (*N* = 141).

**Characteristic**	**Condition**	
	**Expectation confirmation *n* = 77**	**Expectation violation** ***n* = 64**
Age in years, *M* (*SD*)	27.74 (9.20)	26.88 (9.06)
**Sex**, ***n*** **(%)**		
Female	52 (67.5)	46 (71.9)
Male	25 (32.5)	18 (28.1)
**Educational level**, ***n*** **(%)**		
No educational degree	0 (0)	1 (1.6)
Primary education	5 (6.5)	8 (12.5)
Secondary education	47 (61.0)	40 (62.5)
University degree	24 (31.2)	15 (23.4)
Other degree	1 (1.3)	0 (0)
PHQ-9, *M* (*SD*), *range*	10.77 (6.99), 0 to 26	10.08 (6.41), 1 to 25
Rejection sensitivity, *M* (*SD*)	10.52 (5.94)	10.81 (7.00)
Depressive expectation scale: Subscale social expectations, *M* (*SD*)	2.44 (0.92)	2.28 (0.86)
Specific expectations T0, *M* (*SD*)	1.92 (1.14)	2.11 (1.31)
Specific expectations T1, *M* (*SD*)	2.07 (1.30)	3.57 (1.68)
Generalized expectations T0, *M* (*SD*)	3.91 (1.82)	3.90 (1.86)
Generalized expectations T1, *M* (*SD*)	3.97 (1.81)	4.51 (1.66)

M, mean; SD, standard deviation; n, sample size. PHQ-9 Score interpretation: 5 to 9, mild severity; 10 to 14, moderate severity; 15 to 19, moderately severe; 20 to 27, severe.

### Apparatus and stimuli

The experiment was conducted online *via* the survey platform SoSci Survey (46). For the Cyberball game (42), the participants were redirected to the Empirisoft server. We used a customized version of Cyberball 5 for running online (47). The experiment took up to 25 mins.

### Instruction and cover story

At the beginning of the study, the participants were informed that the study aimed to investigate whether friendships could be established *via* online games. They were given the prospect of being able to exchange contact details with their fellow players at the end of the study.

### Pre-experimental measurements

#### Sociodemographics

We assessed the sociodemographic variables, including age, gender, education, and mother tongue.

#### Depressive symptoms

Depressive symptoms were assessed using the module for Major Depressive Disorder (MDD) (PHQ-9) (48) of the German version of the Patient Health Questionnaire (PHQ-D) (49). The PHQ-9 is a nine-item self-reporting questionnaire used to pre-classify depressive disorders. The scale value is calculated from the total of all answers and has a range between 0 and 27. A value in the range of 5 to 9 can be interpreted as Major Depressive Disorder (MDD) with mild severity. A value in the range of 10 to 14 can be interpreted as Major Depressive Disorder (MDD) with moderate severity, a value of 15 to 19 as moderately severe and a value of 20 to 27 as severe. In our sample, the internal consistency for the PHQ-9 was high (Cronbach's α = 0.91).

#### Rejection sensitivity

We used a modified version of the Rejection Sensitivity Questionnaire (RSQ) (50, 51) to assess the individual tendency to perceive and anxiously expect interpersonal rejection. The participants were presented with nine scenarios (e.g., “You are asking your parents or another family member to help you in a difficult financial situation”) and asked to rate how concerned they were to get help, on a Likert scale from 1 (*not concerned at all*) to 6 (*highly concerned*), and how optimistic they were not to be rejected, on a Likert scale from 1 (*highly unlikely*) to 6 (*very likely*). We derived the RSQ scores as suggested in Staebler et al. (51) by calculating a score for each scenario, summing these, and dividing the sum by the number of scenarios.

#### Depressive expectation scale: Subscale social expectations

To access the depression-specific dysfunctional social expectations, we used the subscale “social rejection” of the Depressive Expectation Scale (DES) (34). The DES assesses 25 future-directed, situation-specific expectations. The subscale social-rejection expectation consists of 13 items where participants have to rate to the extent to which they agree to the presented statements (e.g., “When I'm sad or depressed, doing things that I usually enjoy will help me”). Answers can be specified on a Likert scale ranging from 1 (don't agree) to 5 (agree). We built a sum score by dividing the sum of all ratings by the number of items.

### Cyberball

**Figure 2:**
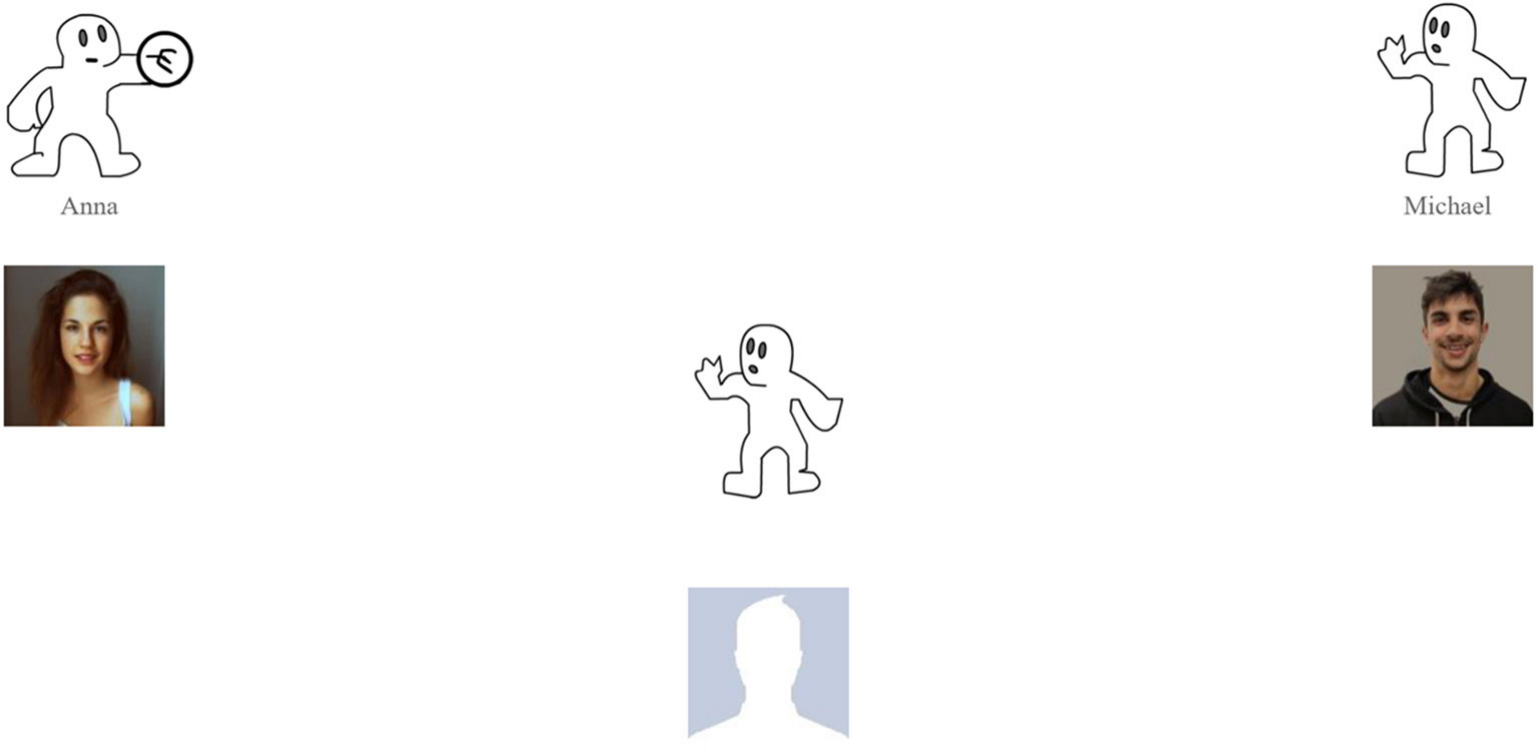
The Cyberball game from the participant's point of view. The co-players are on the right and left sides of the screen. The participant is represented by the empty picture at the bottom of the screen. In this scenario, the computer-generated participant Anna is in the possession of the ball.

In the main part of the experiment, we instructed the participants to play a simple online game with two fellow players, connected over the Internet, involving catching a ball. The process of the game was as follows: when the participant was in possession of the ball, they were required to select the player they wanted to throw the ball to *via* a mouse click. Contrary to what was stated in the instructions, the co-players were not real people, but computer-generated. The ball game from the participant's point of view is shown in Figure 2. During the course of the experiment, the participants played two rounds of the game (with the same co-players), involving 40 throws each. One throw lasted between 6 and 7 s, resulting in a duration of ~4 to 5 min per round.

#### Experimental manipulation of social expectations

Social expectations were manipulated by adjusting the number of throws the participant received from their co-players. During the first round (induction of negative social expectations—exclusion), the participants received the ball only twice at the beginning of the round and were then excluded for the rest of the 40 throws. In the second round, the participants were either excluded one more time (expectation confirmation) or unexpectedly included (expectation violation). Under the inclusion condition, the participants had a 50% chance of receiving the ball at each throw from the other players, and therefore received the ball with a chance of ~33% in total.

#### Manipulation check

First, the participants were asked to rate how ignored and excluded they felt during the game on a Likert scale ranging from 1 (not at all) to 5 (extremely). Then, the participants had to rate the percentage of throws they received, ranging from 0% (no throw) to 100% (every throw). The manipulation check was considered successful when the answer was within a range of ±20% of the actual percentage (so in a range of 13–53% when the actual percentage was 33%, and up to 28% when the actual percentage was 8%). Highly implausible answers in the manipulation check demonstrated that, in those cases, the experimental manipulation of social expectations could not work, which was the reason for us excluding the affected subjects.

#### Reflexive needs

A short version of the reflexive need questionnaire (71, 72) was used to assess the participants' feelings of belonging, self-esteem, control, and meaningful existence during the Cyberball game. Each need was captured using three items, with the participants rating the extent to which the item represented their feelings during the game (e.g., “I felt rejected,” “I felt good about myself,” “I felt powerful”) on a Likert scale from 1 (*not at all*) to 5 (*extremely*). Afterwards, we presented four mood anchors—good, bad, friendly, and unfriendly.

#### Social expectations

We asked the participants to rate their specific and generalized social expectations after each round of the ball game. The items for the situation-specific social expectation were: “If I try to make friends through this online game, I will succeed” and “My co-players seem to like me.” The items for the generalized social expectation were: “If I try to make friends, I will succeed” and “Most people will like me for who I am.” The answers were ranked on a seven-point Likert scale, from 1 (I totally disagree) to 7 (I totally agree). A sum score was calculated for each subarea (specific as well as generalized expectations at the time of Measurements 1 and 2).

### Post-experimental measurements and debriefing

At the end of the study, the participants were asked whether they wanted to exchange email addresses with their co-players (yes or no). Thereafter, they were fully informed about the actual purpose of the study and given the email address of the researcher in case they wanted to ask questions.

### Analysis

Multivariate analyses of variance (MANOVA) were conducted in order to examine potential baseline differences between the two samples (expectation confirmation v. expectation violation) on age, depressive symptoms, rejection sensitivity, depressive social expectations and expectations after exclusion (the first round of Cyberball). Two chi-squared tests of independence were performed in order to examine the distribution of gender and education status. Two linear regressions were performed to predict baseline expectations (specific and generalized) from participants depressive symptoms.

In order to test our main hypotheses concerning social expectations, two 2 (condition: expectation confirmation vs. expectation violation) × 2 (time: after round one of Cyberball vs. after two rounds of Cyberball) factorial ANOVAs were conducted for both types of expectations (specific and generalized). Two moderation analyses were performed to evaluate whether the interaction between condition (expectation confirmation v. expectation violation) and depressive symptoms (PHQ score) significantly predicted generalized and specific social expectation change.

Additional analyses were conducted on feelings associated with social inclusion and exclusion, conceptualized as reflexive needs. Therefore, we conducted another four 2 (condition: expectation confirmation vs. expectation violation) × 2 (time: after round one of Cyberball vs. after two rounds of Cyberball) factorial ANOVAs for each of the four reflexive needs (belonging, self-esteem, control, and meaningful existence). Given the exploratory nature of this study, we set Type-1 error levels at 5%. All analyses were conducted using IBM SPSS Statistics software Version 22 (52). For the moderation analysis we used the PROCESS Macro for SPSS (53).

## Results

### Sample characteristics

Forty-nine participants exited the study at the very beginning, during the introduction or pre-experimental questionnaires. Eleven participants discontinued the study during or after the first round of Cyberball. A total of 155 participants completed the study, of which 14 were excluded because they failed the manipulation check. There were no missing data due to the setup of the study. Therefore, 141 (sample size 77 in the expectation confirmation condition and sample size 64 in the expectation violation condition) were included in the final analysis. The descriptive statistics as well as baseline measurements for the sample are listed in Table 1.

### Baseline differences

As indicated by the MANOVA, the participants from the two conditions (expectation confirmation v. expectation violation) did not differ in terms of age, depressive symptoms, rejection sensitivity, depressive social expectations and situation-specific as well as generalized social expectations at T0, *F*_(6,134)_ = 1.44, *p* = 0.205; η^2^ = 0.060. Also, male and female participants were equally distributed across the two conditions (χ^2^ = 0.311, *p* = 0.577) and the educational status did not differ (χ^2^ = 4.30, *p* = 0.505). Depressive symptoms had a significant influence on specific, *F*_(1,139)_ = 4.45, *p* = 0.037, and generalized, *F*_(1,139)_ = 27.71, *p* < 0.000, social expectations at T0 with lower social expectations at higher values of depressive symptoms.

### Main analysis

#### Changes in specific social expectations

The descriptive statistics are listed in Table 1. The time (T0 vs. T1) x condition (expectation confirmation vs. expectation violation) repeated-measures ANOVA on specific expectations (“If I try to make friends through this online game, I will succeed”) indicated a significant main effect of time (*F*_[1, 139]_ = 64.21, *p* < 0.001, η^2^ = 0.316) with overall higher expectations (*M* = 2.75, *SD* = 1.66) after T1 than after T0 (*M* = 2.00, *SD* = 1.22). There was a significant time x condition interaction (*F*_[1, 139]_ = 41.84, *p* < 0.001, η^2^ = 0.231), which indicated a greater change in specific social expectations in the expectation violation condition compared to the expectation-confirmation condition. Also, the main effect of condition was significant (*F*_[1, 139]_ = 16.80, *p* < 0.001, η^2^ = 108) with higher expectations (*M* = 2.85, *SD* = 1.75) in the expectation violation condition than in the expectation-confirmation condition (*M* = 1.99, *SD* = 1.60).

#### Moderation hypothesis on specific social expectations

**Figure 3:**
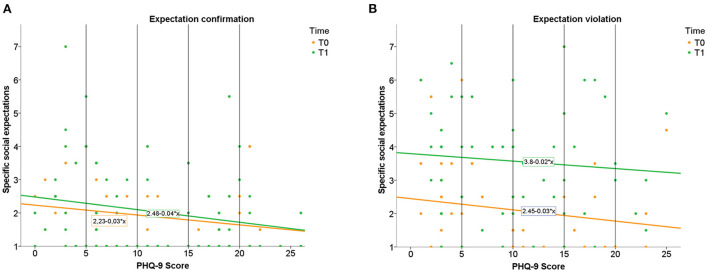
Illustration of the results for specific expectation change. Specific expectations in relation to depressive symptoms (PHQ-9 Score) before and after expectation confirmation **(A)** and expectation violation **(B)**. PHQ-9 Score interpretation (vertical lines): 5 to 9 = mild severity, 10 to 14 = moderate severity, 15 to 19 = moderately severe, 20 to 27 = severe.

A moderation analysis to evaluate whether the interaction between condition (expectation confirmation v. expectation violation) and depressive symptoms (PHQ score) significantly predicted specific social expectation change revealed an overall significant model, *F*_(3,137)_ = 13.92, *p* < 0.001, predicting 23.36% of the variance. Still, results did not show that depressive symptoms moderated the effect condition and change in specific expectations significantly, *F*_(1,137)_ = 18.93, *p* = 0.526. Following Hayes (53) we dropped the interaction from the model. The simple effects model revealed a significant relationship between condition, *B* = 1.05, *p* < 0.001, but not depressive symptoms, *B* < 0.001, *p* = 0.987 and change in specific expectations. Figure 3 visualizes these results.

#### Changes in generalized social expectations

**Figure 4:**
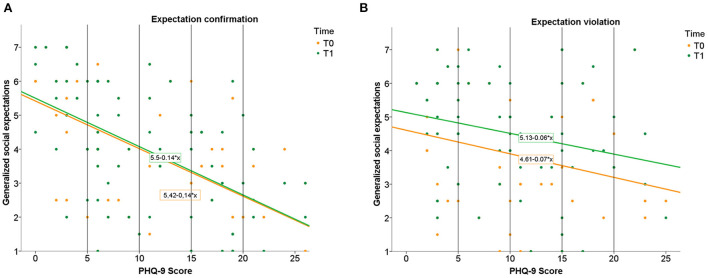
Illustration of the results for generalized expectation change. Generalized expectations in relation to depressive symptoms (PHQ-9 Score) before and after expectation confirmation **(A)** and expectation violation **(B)**. PHQ-9 Score interpretation (vertical lines): 5 to 9 = mild severity, 10 to 14 = moderate severity, 15 to 19 = moderately severe, 20 to 27 = severe.

With one exception, the pattern of results for the generalized expectation (“If I try to make friends, I will succeed” and “Most people will like me for who I am”) was similar to the one for specific expectation change. The time (T0 vs. T1) x condition (expectation confirmation vs. expectation violation) repeated-measures ANOVA on generalized expectations indicated a significant main effect of time (*F*[1,139] = 16.14, *p* < 0.001, η^2^ = 0.104) with overall higher expectations (*M* = 4.21, *SD* = 1.76) after T1 than after T0 (*M* = 3.90, *SD* = 1.83). There was a significant time x condition interaction (*F*[1,139] = 10.99, *p* = 0.001, η^2^ = 0.073), which indicated greater change in generalized social expectations in the expectation violation condition compared with the expectation-confirmation condition. In contrast to the specific-expectation change, the main effect of condition was non-significant (*p* = 0.364). Figure 4 shows the results for generalized expectation change.

#### Moderation hypothesis on generalized social expectations

Similar to the results of specific expectations, the overall model was significant, *F*_(3,137)_ = 13.92, *p* = 0.0137, even though results did not show that depressive symptoms moderated the effect condition and change in generalized expectations significantly, *F*_(1,137)_ = 0.174, *p* = 0.677. The simple effects model revealed a significant relationship between condition, *B* = 0.552, *p* = 0.001, but not depressive symptoms, *B* = 0.002, *p* = 0.868 and change in generalized expectations.

### Additional analysis: Changes in reflexive needs

The descriptive statistics for the four basic reflexive needs on the two measurement points are listed in Table 2. The time (T0 vs. T1) x condition (expectation confirmation vs. expectation violation) repeated-measures ANOVA on each scale of the need-threat scale (belonging, self-esteem, meaningful existence, and control) revealed four main effects for time and condition (all *p*-values < 0.001). These results indicate an overall greater need fulfillment at the second measurement point compared with the first measurement point, as well as higher scores in the fulfillment of needs in the expectation violation condition compared with the expectation-confirmation condition. Also, all four interaction effects of time and condition were significant (all *p*-values < 0.001). As expected, need fulfillment at the second measurement point was greater in the expectation violation group than in the expectation-confirmation group. All alpha levels would survive Bonferroni-corrections.

**Table 2:** Additional analysis: comparison of the two experimental conditions regarding the self-rating on reflexive needs (*N* = 141).

**Need and time of measurement**	**Condition**	
	**Expectation confirmation** ***N = 77***	**Expectation violation** ***N = 64***
	***M* (*SD*)**	***M* (*SD*)**
Belonging T0	1.97 (1.01)	2.06 (1.07)
Belonging T1	2.07 (1.15)	4.11 (0.93)
Self-esteem T0	2.01 (0.94)	2.38 (1.10)
Self-esteem T1	2.15 (1.01)	3.72 (0.86)
Meaningful existence T0	2.42 (1.24)	2.56 (1.20)
Meaningful existence T1	2.24 (1.30)	4.33 (0.93)
Control T0	1.51 (0.77)	1.68 (0.75)
Control T1	1.49 (0.77)	2.47 (0.92)

M, mean; SD, standard deviation; n, sample size; T0, after one round of Cyberball; T1, after two rounds of Cyberball.

## Discussion

Our experiment was conducted in order to evaluate participants' reactions regarding social expectations to unexpected social inclusion. We experimentally manipulated the degree of social inclusion by varying the number of throws participants received from their two co-players in an online ball game. In the first round, all participants experienced social exclusion by not receiving the ball from their co-players apart from twice at the beginning (induction of negative social expectations). In the second round, the participants were either once more excluded (expectation confirmation) or were included by receiving the ball as often as their co-players did (expectation violation). The central question was whether unexpected social inclusion changed their generalized and specific social expectations. Additional analyses were performed to investigate how expectation violation affected feelings associated with social inclusion and exclusion, conceptualized as four reflexive needs.

We found that unexpected social inclusion in the cyberball paradigm led to a significant shift of specific as well as generalized social expectations. Contrary to what was expected, we found that depressive symptoms did not moderate expectation change after positive expectation violations. However, as hypothesized, depressive symptoms moderated social expectations (e.g., that their co-players liked them) at T0. Additional analyses were conducted on the four reflexive needs. The results show, that according to our hypothesis, unexpected social inclusion resulted in a significant change of all four needs.

First of all, these results confirm that Cyberball is a suitable tool for manipulating feelings related to social inclusion and exclusion. To our knowledge, our study is the first to show that Cyberball is also suitable for inducing *and changing* expectations that arise in social situations (social expectations). Furthermore, our findings are in line with studies showing that people with Major Depressive Disorder (MDD) have overall more negative social expectations (34) and are more sensitive to social exclusion and ambiguous social stimuli than non-depressed people (44, 45). Given the literature on cognitive immunization (32, 54), the non-significant relationship between depressive symptoms and change of social expectations is worthy of an in-depth discussion.

The questions that arise when interpreting the unexpected change in expectation also in the presence of depressive symptoms is: what are the circumstances in our experiment that made that change possible, and which group of patients and which situations are these conclusions valid and useful for? As hypothesized in the ViolEx Model 2.0 (30), it can be assumed that the high level of expectation violation that was used in our experiment supported the change in expectations. Furthermore, it can also be assumed that the explicit appraisal of the social inclusion after each round (manipulation check: “Please rate what percentage of the throws you received”) increased the valence of the expectation violation, and that the repeated verbalization of expectations increased the expectation violation effects (30, 55). In total, the setup of our experiment was similar to an expectation-focused intervention (35) or behavioral experiment in cognitive behavioral therapy in which a situation is specifically sought out to test the validity of a dysfunctional expectation (35) or a dysfunctional belief (56, 70). Behavioral experiments and expectation-focused interventions are carried out in order to disconfirm beliefs and expectations, thereby changing them, and should thus lead to symptom reduction (35, 57), which was, in fact, observed in our experiment (participants reported fewer depression-typical beliefs and symptoms after unexpected social inclusion). In line with a recent study (58), we can conclude that abnormal expectation violation effects in Major Depressive Disorder (MDD) are only found under specific circumstances, but not in general. Some findings suggest that, under certain conditions, the situational processing of people Major Depressive Disorder (MDD) can be even more accurate than that of non-depressed people (59, 60). In particular, this seems to affect the so called “illusion of control,” which means that non-depressed samples tend to overestimate the degree of control they have over an outcome (61, 62, 73).

Reflexive needs consistently increased after expectation violation (Table 2), but did not show a consistent pattern after expectation confirmation. Descriptive statistics reveal that after expectation confirmation, the mean value of two needs marginally increased in two needs (belonging and self-esteem), while they decreased in two other needs (meaningful existence and control. On a descriptive level, this trend can lead the hypothesis that social rejection is substantially painful on an emotional level (38, 63) and hinders to engage in the above mentioned “optimism bias.”

### Strength and limitations

By using the Cyberball paradigm as the main part of our study, we ensured high comparability and easy replication of the results because it is one of the most often-used paradigms for manipulating interpersonal acceptance and exclusion, being accessible *via* open access. This made it possible to easily replicate the experiment and to adjust various points (e.g., the proportion of positive reinforcement in terms of the frequency at which the participant receives the ball). Still, the paradigm has the disadvantage that the social situation created is somewhat artificial.

Considering the further limitations of our study, it would have been helpful to have included expert clinical assessments of the depressive symptomatology, even though a meta-analysis has reported that the PHQ-9 has a sensitivity of 80% and a specificity of 92% (64). Also, due to ethical reasons, we did not assess the current or past psychotherapeutic or pharmacological treatments in order to avoid the online assessment of sensitive information. This is of particular relevance, as numerous studies have shown that the serotonergic system plays an important role in the modulation of human social behavior (65, 66). Nevertheless, the DES, designed to measure depression-typical expectations (34), and the RSQ (50, 51) achieved corresponding results in our sample, which is an indication of a reliable sampling procedure. Unfortunately, the group size for each condition (expectation confirmation vs. expectation violation) differed. However, the analysis revealed no baseline differences between the two samples.

We continued with the limitations of previous studies by assessing expectations using only a few items, which may not be sufficient to capture every individual's implicit and explicit expectations concerning social situations (67). Also, our assessment did not include any biological variables or any chance for the participant to behave in a different way than that provided by the experiment. Therefore, we cannot draw any conclusions about the participants' self-motivated behaviors.

### Clinical implications and directions for future research

First of all, our results are an experimental confirmation that a change in negative to positive social expectations is possible in a non-clinical population despite the presence of depressive symptoms. Expectation violation did not only lead to changes in specific expectations, but also to changes in generalized expectations. Changing generalized expectations is a major goal of therapeutic interventions, such as the Cognitive Behavioral Analysis System of Psychotherapy (CBASP), in which a key element—the situation analysis—explicitly aims to generalize the results of cognitive restructuring and role playing (29, 68). In our study, the repeated verbalization of expectations might have contributed to this change, whereas other studies have found more resistant negative expectations in people with Major Depressive Disorder (MDD). Also, concerning the feelings associated with inclusion and exclusion, we obtained a positive shift in the expectation violation group, irrespective of the prevalence of depressive symptoms. This fact not only confirms the interrelationship between reflexive human needs and expectations, but also that it is possible to change these feelings in a sample with depressive symptoms. For therapeutic practice, it can be deduced that experiencing new, positive experiences can counteract depressive symptoms.

In order to further differentiate between the results, it is necessary to adapt the paradigm to a “real-life” situation with real co-players in order to overcome the limitation of the reduced ecological validity of the Cyberball paradigm. Another valid next step would be to apply the paradigm to a clinical population, especially to chronically depressed patients, in whom interpersonal deficits seem to be particularly central (29). It may be that chronically depressed people will not succeed in changing their expectations.

## Conclusion

Our results speak in favor of our experimental setup being suitable for examining the change of social expectations and need fulfillment in a non-clinical sample. As proposed in the ViolEx Models, specific and generalized social expectations are changeable and increase following expectation violation created by better-than-expected social inclusion. In our non-clinical sample, depressive symptoms did not hinder expectation change after positive expectation violation. The requirements to apply the paradigm on a clinical sample are given.

## Data availability statement

Due to privacy rules, the analyzed data are not openly available as conclusions about individual participants cannot be ruled out. Requests to access the datasets should be directed to rosa-marie.groth@uni-marburg.de.

## Ethics statement

The studies involving human participants were reviewed and approved by Local Ethics Committee of Philipps University Marburg. The participants provided their written informed consent to participate in this study.

## Author contributions

R-MG was the principal investigator, in terms of study design, implementation, data collection, and analysis, was mainly involved in the development of the manuscript, and has agreed to be responsible for all aspects of the work and to ensure that issues relating to the accuracy or integrity of any part of the work are adequately investigated and resolved. WR supervised the conception of the study, critically revised the manuscript, has consented to the publication of the manuscript, and has agreed to be responsible for all aspects of the work and to ensure that issues relating to the accuracy or integrity of any part of the work are adequately investigated and resolved. All authors contributed to the article and approved the submitted version.

## Conflict of interest

The authors declare that the research was conducted in the absence of any commercial or financial relationships that could be construed as a potential conflict of interest.

## Publisher's note

All claims expressed in this article are solely those of the authors and do not necessarily represent those of their affiliated organizations, or those of the publisher, the editors and the reviewers. Any product that may be evaluated in this article, or claim that may be made by its manufacturer, is not guaranteed or endorsed by the publisher.
